# Modeling Alzheimer’s disease in primary neurons reveals DNA damage response coupled with MAPK-DLK signaling in wild-type tau-induced neurodegeneration

**DOI:** 10.21203/rs.3.rs-2617457/v1

**Published:** 2023-03-07

**Authors:** Sanming Li, Ethan R. Roy, Yanyu Wang, Trent Watkins, Wei Cao

**Affiliations:** Department of Anesthesiology, Critical Care and Pain Medicine, McGovern Medical School, University of Texas Health Science Center at Houston, Houston, TX 77030, USA;; Department of Anesthesiology, Critical Care and Pain Medicine, McGovern Medical School, University of Texas Health Science Center at Houston, Houston, TX 77030, USA;; Department of Anesthesiology, Critical Care and Pain Medicine, McGovern Medical School, University of Texas Health Science Center at Houston, Houston, TX 77030, USA;; Department of Neurosurgery, Baylor College of Medicine, Houston, TX, USA. Current address: Department of Neurology, University of California at San Francisco, San Francisco, CA 94158 USA; Department of Anesthesiology, Critical Care and Pain Medicine, McGovern Medical School, University of Texas Health Science Center at Houston, Houston, TX 77030, USA;

**Keywords:** Alzheimer, Tauopathy, Neurodegeneration, DNA damage, MAP kinase, DLK, Axonal degeneration

## Abstract

**Background:**

Alzheimer’s disease (AD) is the most prevalent form of neurodegeneration. Despite the well-established link between tau aggregation and clinical progression, the major pathways driven by this protein to intrinsically damage neurons are incompletely understood.

**Methods:**

To model AD-relevant neurodegeneration driven by tau, we overexpressed wild-type human tau in primary mouse neurons and characterized the subsequent cellular and molecular changes. RNAseq profiling and functional investigation were performed as well. A direct comparison with a mutant human tau was conducted in detail.

**Results:**

We observed substantial axonal degeneration and cell death associated with wild-type tau, a process accompanied by activated caspase 3. Mechanistically, we detected deformation of the nuclear envelope and increased DNA damage response in tau-expressing neurons. Gene profiling analysis further revealed significant alterations in the mitogen-activated protein kinase (MAPK) pathway; moreover, inhibitors of dual leucine zipper kinase (DLK) and c-Jun N-terminal kinase (JNK) were effective in alleviating wild-type human tau-induced neurodegeneration. In contrast, mutant P301L human tau was less toxic to neurons, despite causing comparable DNA damage. Axonal DLK activation induced by wild-type tau potentiated the impact of DNA damage response, resulting in overt neurotoxicity.

**Conclusions:**

We have established a cellular tauopathy model highly relevant to AD and identified a functional synergy between DNA damage response and the MAPK-DLK axis in the neuronal degenerative process.

## Background

Microtubule-associated protein tau (encoded by *MAPT* gene) is centrally involved in the pathogenesis of Alzheimer’s disease (AD) and a group of neurodegenerative disorders coined as “tauopathies”, which display a hallmark of excessive tau aggregation in the brain. Tau is most abundantly expressed in neurons, where it primarily associates with and stabilizes microtubules. Besides this role, tau has been shown to fulfill biological functions in regulating key neuronal processes, including axonal transport, cytoskeletal dynamics, synaptic transmission, nuclear transport, protein translation, mitochondrial function, and metabolism [1, 2]. The complex biology of this intrinsically disordered protein entails multiple isoforms generated from alternative splicing of a single *MAPT* gene, a plethora of post-translational modifications at various sites of the protein, and the discrete roles played by tau in different cellular compartments [3–7].

AD distinguishes from other tauopathies by presentation as a secondary tauopathy. In primary tauopathy conditions, such as frontotemporal lobar degeneration (FTLD), Pick disease, progressive supranuclear palsy (PSP), corticobasal degeneration (CBD), and chronic traumatic encephalopathy (CTE), a large number of rare *MAPT* mutations predispose the development of tau pathology in the central nervous system (CNS)[5]. In contrast, the vast majority of AD patients possess non-mutated *MAPT* while developing abundant amyloid plaques in conjunction with neurofibrillary tangles (NFT) that contain insoluble tau aggregates in their brains [8, 9]. Through studying animal and cell culture models expressing mutated *MAPT*, many groups over the years have elucidated the pathogenic potential of FTLD-associated mutant tau [10–12]. However, a critical question that remains to date is whether the pathogenic role of tau in AD is in any way dissimilar from that in primary tauopathy. If so, the findings from the mutant tau studies might be inadequate to guide the therapeutic development for AD.

Elevated tau protein level is causally linked to disease pathogenesis; in particular, intraneuronal accumulation of aggregated tau closely correlates with the clinical progression of AD as well as other tauopathies [13, 14]. In rodent brains, neuronal overexpression of mutant *MAPT* is sufficient for the development of NFT pathology and the onset of neurodegeneration, leading to the successful construction of numerous tauopathy disease models [12, 15]. *In vitro*, however, tau overexpression has been mostly carried out in immortalized non-neuronal cells, such as HEK293 and HELA cells, to facilitate studies on the tau interactome, transcriptomic influence by tau, or self-aggregation and seeding properties of tau [16–18]. Surprisingly little is known about the functional impact of increased intraneuronal tau on differentiated primary neurons. Consequently, the major cellular pathways that govern tau-mediated neuropathology relevant to AD remain poorly understood.

To investigate AD-relevant tau-dependent neurodegeneration, we developed a cellular tauopathy model by overexpressing human tau in mouse primary neurons. This system afforded us to comprehensively assess neuronal intrinsic responses to full-length wild-type (WT) tau, and enabled a direct comparison with an FTLD-associated mutant form. Remarkably, we have detected heightened neurotoxicity triggered by WT tau and obtained insights on two major signaling pathways that promote the degenerative process that is pertinent to AD.

## Methods

### Animals

C57BL/6J mice bred from line originally obtained from the Jackson Laboratory were used in this study. Mice were housed in groups of 2–3 per cage under conventional or specific pathogen-free conditions and standard light/dark cycle. Both male and female neonates were used in experiments. All animal procedures were performed in accordance with NIH guidelines. The experimental protocols were approved by the Animal Welfare Committee of The University of Texas Health Science Center at Houston or Institutional Animal Care and Use Committee of Baylor College of Medicine.

### Generation of AAV vectors

Recombinant AAV1/2 vectors containing the longest tau isoform (2N4R) of human *MAPT* cDNA of wild-type and mutant P301L under human SYN1 promoter were used [19]. To construct a control vector, a stop codon arisen from single nucleotide mutation was generated at the N-terminus of the *MAPT* sequence. The detailed sequences were provided in supplemental data 4. Recombinant AAV stocks were produced by Gene Vector Core at Baylor College of Medicine. Vector genomes were titrated by quantitative PCR and purity validated by SDS-PAGE. The titer of 4 × 10^9^ GC/ml was selected for neuronal cell infection.

### Primary cultures of mouse cortical and hippocampal neurons

Primary neurons were harvested and cultured as previously described [20]. Briefly, P0 pups of mixed sexes were decapitated into ice-cold dissection buffer (1x HBSS supplemented with 10 mM HEPES, pH 7.5, 0.6% glucose, 20 U/ml penicillin, 20 μg/ml streptomycin). Forebrain hemispheres were dissected and stripped of meninges. The tissues of cortex with hippocampus were isolated using a dissecting microscope and digested in trypsin at 37°C for 15 minutes with gentle swirling. Following addition of 500 μl trypsin (2.5%), 400 μl soybean trypsin inhibitor (1 mg/ml) and 100 μl DNase I (1%) were then added. After tissue pieces decanted off, the supernatant was removed and replaced with 2 ml of DMEM and 20 μl DNase I (1%). The digested tissues were triturated with a P1000 pipette tip 8–10 times. After allowing the remaining pieces to settle, the supernatant was collected into a fresh tube for centrifuging at 1200 rpm for 5 min. The cell pellet was resuspended with 5ml of DMEM and then centrifuged again. After carefully removed the supernatant, the dissociated cells were resuspended in 2 ml completed neuronal culture media (Neurobasal medium supplemented with 2% B27, 0.5 mM L-glutamine, 40 U/ml penicillin, 40 μg/ml streptomycin), and passed through a 70 μm filter. The single suspended cells were plated into poly D-lysine-coated culture plates or glass coverslips (1–2 × 10^5^ cells/cm^2^). Cells were maintained in incubators at 37°C, 5% CO_2_, and half the culture medium was replaced every 5–7 days.

### LDH cytotoxicity assay

50 μL of culture media was analyzed by the standard procedure provided by the manufacturer (Cat# C20301, CyQUANT^™^ LDH Cytotoxicity Assay, Thermo fisher, USA). The absorbance by excitation of 560 nm and emission of 590 nm was measured on the SpectraMax^®^ ABS Microplate Reader (Molecular Devices, USA).

### Neuronal culture treatments

10 mM pan-caspase inhibitor Z-VAD-FMK (Cat# HY-16658B, MCE), or 2 mM JNK inhibitor D-JNKI-1(JNKi) (Cat# HY-P0069, MCE), or 250nM DLK inhibitor GNE-3511 (DLKi) (Cat# HY-12947, MCE) was added to the cultured media at Day 5 post-AAV infection. Equal volume of DMSO was added in vehicle control samples. The cell density and LDH analysis was performed after 72–96 hours treatment. The dose of the inhibitors was chosen to minimize cytotoxicity to the cultured neurons. 100 nM colchicine (Cat# HY-16569, MCE) or 2 mM etoposide (HY-13629, MCE) was added singly or together to the cultured media at day 7–14 post-AAV infection.

### Immunofluorescent staining

The cultured neurons on the coverslips were fixed with 4% paraformaldehyde (Santa Cruz, cat# sc-281692) for 20 minutes at 4°C. After washing with 1x PBS for 3 times, the cells were incubated in 0.2% Triton X-100 for 20 minutes. After rinsing the cells with PBS twice and pre-incubating them with a blocking buffer of 10% normal donkey serum (Cat# S30–100ML, Millipore) and 0.5% Triton X-100 in PBS for 1 h, the cells were incubated with primary antibodies anti-HT7 (Cat# MN1000, Invitrogen), Total tau (Cat# T6402, Sigma), PHF1 (provided by Dr. Peter Davies), AT8 (Cat# MN1020, Invitrogen), CP13 (provided by Dr. Peter Davies), AT180 (Cat# MN1040, Invitrogen), Tau pS262 (Cat# 44–750G, Invitrogen), MC1 (provided by Dr. Peter Davies), Phospho-Neurofilament H (pNF-H) (Cat# 801601, Biolegend), β-Tubulin III (Cat# 801201, Biolegend), Synaptophysin (Cat# AF5555, R&D), PSD-95 (Cat# 51–6900, Invitrogen), HP1α (Cat# 2616, Cell Signaling Technology), Phospho-Histone H2A.X (pH2AX)(Cat# 9718, Santa Cruz), Lamin A/C (Cat# sc-376248, Santa Cruz), Phospho-p53(Ser15) (Cat# 9284, Cell Signaling Technology), Active Caspase 3 (Cat# 9664, Cell Signaling Technology), Puma (Cat# A3752, Abclonal), NeuN (Cat# ABN91, Millipore), c-Jun (Cat# A2046, Abclonal), Phospho-JNK (p-JNK) (Cat# 4668, Cell Signaling Technology), Phospho-c-Jun (p-c-Jun) (Ser73) (Cat# 3270, Cell Signaling Technology), p-c-Jun (Ser63) (Cat# AP0105, Abclonal), at 4°C overnight. After 3 separate 10 minutes washes with PBS, they were then incubated with fluorescent secondary antibodies diluted in blocking buffer for 1 hour at room temperature. Following 3 additional PBS washes for 10 minutes each, the cells were counterstained with DAPI, mounted with mounting medium, and photographed using the confocal laser scanning microscopy (Leica, Germany) or EVOS fluorescence microscopy (Life Technologies, USA).

### Immunoblotting

Total protein from cultured neurons were extracted with a cold RIPA lysis buffer composed of protease and phosphatase inhibitor mixtures. The protein concentration was quantified using the BCA assay. The tissue or cell lysate with equal amounts of proteins were subjected to electrophoresis on 8 to 15% SDS-PAGE and then electrophoretically transferred to a PVDF membrane. After blocking in 5% milk for 1 h, the membranes were incubated with primary antibodies Total tau, HT7, AT8, CP13, AT180, PHF1, p-c-Jun (Ser73), Synaptophysin, PSD-95, p-JNK, pH2AX, Active Caspase 3, γ-tubulin (Cat# T6557, Sigma), β-Actin (Cat# sc-47778, Santa Cruz) overnight at 4°C. After three washes with Tris-buffered saline containing 0.05% Tween 20 for 10 minutes each, the membranes were incubated with fluor-conjugated Donkey anti-Mouse IgG (Cat# 926–68072, LI-COR), Donkey anti-Rabbit IgG (Cat# 926–68073, LI-COR), or Donkey anti-Goat IgG (Cat# 926–68074, LI-COR) for 1 h. After three times of washing, the signals were visualized by on a LI-COR Odyssey blot imager or Bio-Rad ChemiDoc^™^ Imagers. The band intensities were normalized to the corresponding value of γ-Tubulin or β-actin expression as a loading control. For tau oligomers detection, the method from Dr. Kayed’s lab was followed [21] and a pre-cast NuPAGE 4–12% Bis-Tris Gels for SDS-PAGE (NP0335BOX, Invitrogen) was used for oligomeric tau detection.

### Quantitative real-time RT-PCR analysis

Total RNA was preserved in TRIzol solution and extracted with Direct-zol RNA Microprep Kits (Cat# R2062, ZYMO Research). About 200ng-1ug of RNA was used to generate cDNA by reverse transcription using iScript Reverse Transcription Supermix reagent (Cat# 170–8840, Bio-Rad). qRT-PCR was performed using iTaq Universal SYBR Green Supermix (Cat# 172–5124, Bio-Rad) on a CFX384 Touch Real-Time PCR Detection System. The primers used to amplify specific gene products are listed in Table S1. The results of relative quantitative PCR were analyzed using the comparative threshold cycle (Ct) method and normalized to *Hprt1* expression as an endogenous reference.

### RNA-seq

Total RNA was extracted with Direct-zol RNA Microprep Kits. Novogene Co. (CA, USA) performed mRNA sequencing and data analysis. Basically, the RNA quality was evaluated as follows: RNA integrity number > 7.0 and 28S:18S ratio > 1.8. Messenger RNA was purified from total RNA using poly-T oligo-attached magnetic beads for library construction. The library cDNA was subjected to paired-end sequencing with a pair end 125-base pair reading length on an Illumina HiSeq 2500 sequencer (Illumina, San Diego, CA, USA). For quantification of gene expression level, featureCounts v1.5.0-p3 was used to calculate the reads numbers mapped to each gene. And then FPKM of each gene was determined based on the length of the gene and reads count mapped to this gene. Differential expression analysis was performed using the DESeq2 R package (1.20.0). The P-values were adjusted using the Benjamini and Hochberg’s approach for controlling the false discovery rate. Genes with an adjusted P-value < = 0.05 found by DESeq2 were considered as differentially expressed. ClusterProfiler R package was used to test the statistical enrichment of differential expression genes in KEGG pathways. The local version of analysis tool (http://www.broadinstitute.org/gsea/index.jsp), GO, KEGG, Reactome, DO and DisGeNET data sets were used for Gene Set Enrichment Analysis (GSEA).

### Quantification of axon degeneration and neurofilament fragments in vitro

The cultured neurons were immuno-stained with β-tubulin III antibody to visualize the microtubule structure, or with Phospho-Neurofilament H (pNF-H) to visualize the neurofilament. For per image, the total number of spheroids on the microtubule and neurofilament fragments was counted by ImageJ software. Then the number was normalized by dividing to the area (pixels) of β-tubulin III or pNF-H positive expression. More than 6 independent areas from 3 individual slides were analyzed for each experimental condition.

### Thioflavin S staining

Fresh Thioflavin S (ThioS) solution was prepared by dissolving 1 g of ThioS (Cat# T1892, Sigma) in 100ml 80% ethanol, and stirring overnight at 4°C, and filtering for final use. The fixed cells were rinsed in PBS, then transferred to solution containing 0.0002% thioflavin S in PBS for 8 mins, rinsed in 40% ethanol in PBS twice for 2 mins, followed by two rinses in PBS, then mounted on slides.

### Quantification and statistical analysis

Statistical analysis was performed using GraphPad Prism 9 (GraphPad Software, San Diego, CA, USA). All data in bar plots are presented as mean ± SEM. Data are representative of two or three independent experiments. Unless otherwise noted, differences between two groups were analyzed by two-tailed Student’s t-tests, and differences between three or more groups were analyzed by one-way ANOVA with Tukey’s multiple comparisons test, as indicated in figure legends. The rate of nuclear envelop invagination between groups was compared by Fisher’s exact test. p < 0.05 was considered statistically significant (noted as ∗ P < 0.05, ∗∗P < 0.01, ∗∗∗P < 0.001 in plots), and those over 0.05 were considered non-significant (“ns”, or numerical P values listed in certain plots). All n values are listed in figure legends for each respective plot. All micrographs shown are images representative of multiple replicates as indicated.

## Results

### Neuronal wild-type human tau overexpression prompts axonal and neuronal degeneration

**Figure 1:**
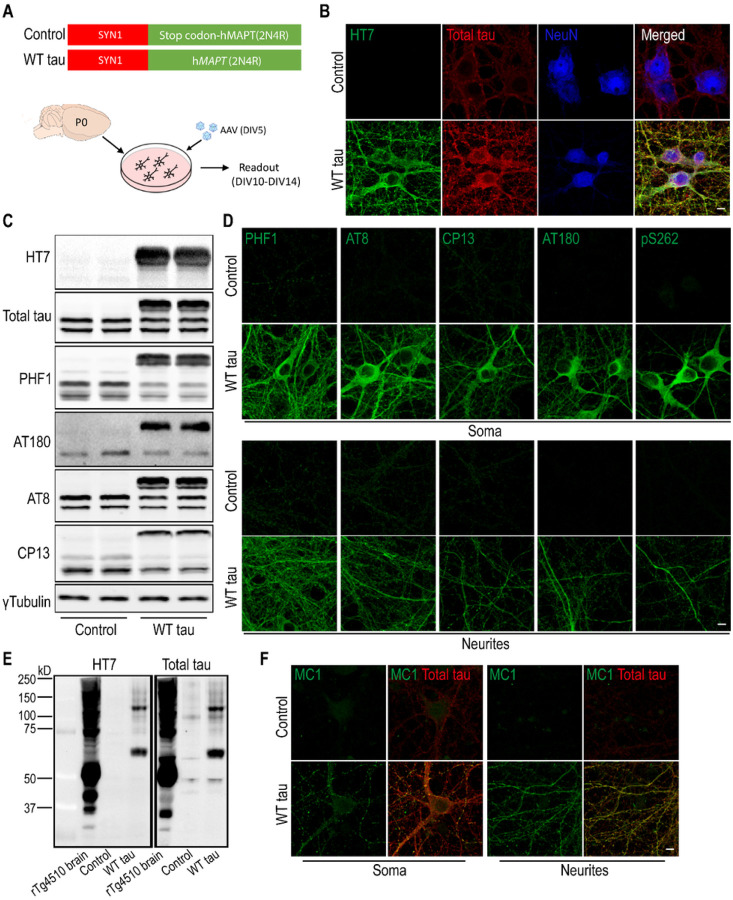
Expression of human wild-type tau protein in mouse primary neurons. **(A)** Mouse cortical neurons were cultured *in vitro*. At DIV5, cells were infected either with an AAV vector containing full-length human *MAPT* sequence (2N4R), or a control vector with an inserted stop codon at the N-terminus of the *MAPT* sequence. **(B)** Immunofluorescent staining of tau in neurons (NeuN positive) at day 7 post-infection. The scale bar represents 5 μm. **(C)**Western blot results of tau protein expression in control and WT tau groups. **(D)**Phosphorylated tau visualized in somas and neurites of neurons. The scale bar represents 5 μm. **(E)** Tau oligomerization assessed by western blot. Brain lysate of rTg4510 tauopathy mice was used as positive control. **(F)** Presence of misfolded and aggregated tau visualized with antibody MC1. The scale bar represents 5 μm.

To enable human tau expression, primary mouse neurons were infected at DIV5 with an AAV vector containing the human WT *MAPT* gene (2N4R) under the control of the human *SYN1* promoter (Fig 1A). Full-length human tau (hTau) protein was produced in the neurons in a time-dependent manner and reached an estimated ratio of 2:1 relative to endogenous mouse tau protein (Fig S1A-B). Neuronal hTau expression was further confirmed by immunostaining with a hTau-specific antibody (clone HT7) and a polyclonal antibody that recognizes total tau protein (both human and mouse) (Fig 1B). In addition, we detected hTau phosphorylation at multiple amino acid positions (Ser396/Ser404 (PHF-1^+^), Thr231 (AT180^+^), Ser202/Thr205 (AT8^+^), and Ser202 (CP13^+^)) (Fig 1C), which were localized in the soma as well as neurites of the *MAPT*-transduced neurons (Fig 1D). Hyperphosphorylation can promote tau aggregation and NFT formation [1, 2]. We detected high molecular weight multimeric species of hTau protein by western blot (Fig 1E) and upregulation of a conformational epitope of aggregated hTau (Fig 1F); however, no signal was obtained by Thioflavin S staining (Fig S1C). These findings suggest that WT full-length *MAPT* overexpression in primary neurons leads to human tau hyperphosphorylation and aggregation in the absence of NFT.

**Figure 2:**
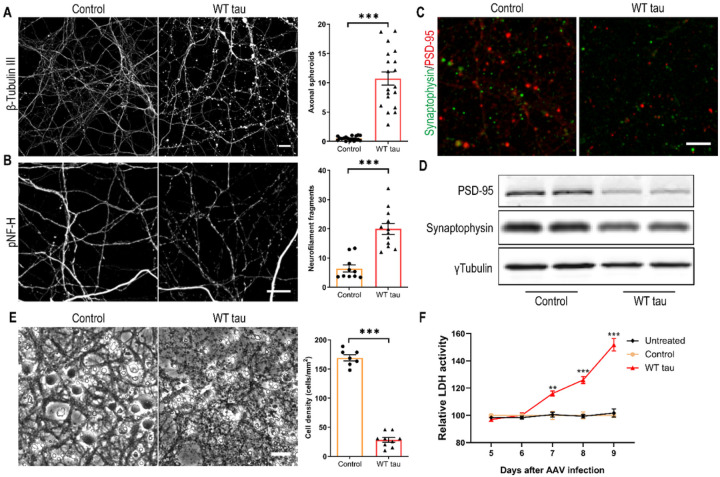
Axonal degeneration and cell death in WT hTau-expressing neurons. **(A)** Representative images of β-tubulin III staining in control and WT tau groups at day 7 post-infection and quantification of axonal spheroids. The scale bar represents 10 μm. n=18, 19 individual images in control, WT tau groups, respectively. **(B)** Representative images of phosphorylated neurofilament heavy chain (pNF-H) staining in control and WT tau groups at day 7 post-infection and quantification of neurofilament fragments. The scale bar represents 10 μm. n=10, 12 individual images in control, WT tau groups, respectively. **(C)** Representative images of PSD-95 and synaptophysin co-staining in control and WT tau groups. The scale bar represents 5 μm. **(D)** Western blot results of PSD-95 and synaptophysin expression in control and WT tau groups. **(E)** Representative cell images in control and WT tau groups at day 9 post-infection and quantification of cell density. The scale bar represents 25 μm. n=7, 9 individual images in control, WT tau groups, respectively. **(F)** Kinetic analysis of LDH activity in the culture media from untreated, control and WT tau groups. n=6 wells/group in each time point.

Several days after AAV infection, WT hTau+ neurons started to display morphological changes indicative of degeneration. By day 7 post-infection, we observed gross axonal degeneration manifested by the development of numerous axon swelling or spheroids (Fig 2A) and the accumulation of fragmented neurofilaments in culture (Fig 2B). In conjunction, synaptic markers were significantly reduced (Figs 2C and 2D). By day 9 post-infection, neuronal density was decreased by > 80% in WT hTau+ culture (Fig 2E). Moreover, by assaying the culture medium longitudinally, we detected a time-dependent escalation of lactate dehydrogenase (LDH) release, corroborating a remarkable neurotoxic phenotype of intracellular WT human tau in primary neurons (Fig 2F).

### Caspase 3 activation is involved in WT hTau-dependent neurodegeneration

**Figure 3:**
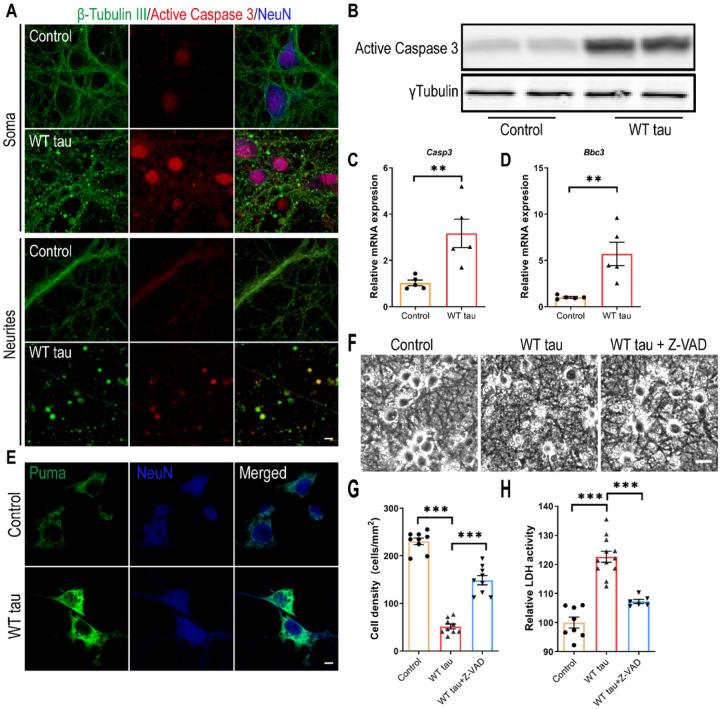
Caspase 3 activation in WT hTau-expressing neurons. **(A)** Representative images of β-tubulin III and active caspase 3 co-staining in somas and neurites at day 7 post-infection. The scale bar represents 5 μm. **(B)** Active caspase 3 expression assessed by western blot. **(C)** Relative expression of *Casp3* mRNA at day 7 post-infection. n=5 samples/group. **(D)** Relative expression of *Bbc3* mRNA at day 7 post-infection. n=5 samples/group. **(E)** Representative images of Puma staining in control and WT Tau-expressing neurons. The scale bar represents 5 μm. **(F)** Representative cell images in control or WT tau groups with or without pan-caspase inhibitor Z-VAD-FMK treatment. The scale bar represents 25 μm. **(G)** Quantification of cell density 72 hrs after the treatment as shown in **F**. n=9 individual images/group. **(H)** Relative LDH activity 72 hrs after the treatment as shown in **F**. n=8, 12, 6 wells in control, WT tau, WT tau + Z-VAD groups, respectively. Data are presented as mean ± SEM. Statistical significance was determined using unpaired, two-tailed Student’s t test in C and D, or one-way ANOVA with Tukey’s multiple comparisons test in G and H.

To investigate the neurotoxicity elicited by WT hTau, we first examined apoptosis, a programmed cell death pathway that has been implicated in the loss of neurons in AD [22]. Apoptotic death involves the function of caspases, among which caspase 3 activation serves as a central effector [23]. In WT hTau^+^ neurons, we detected increased abundance of cleaved caspase 3, the signal of which was present both inside the nuclei and in the neuritic areas rich with axonal spheroids (Figs 3A and 3B). Consistently, WT hTau^+^ neurons expressed more *Casp3* mRNA (Fig 3C). PUMA, encoded by the *Bbc3* gene, is critically involved in the intrinsic apoptotic signaling pathway [24]. We detected elevated expression of both mRNA and protein products of *Bbc3* in neurons with WT hTau (Figs 3D and 3E). To test the importance of caspase activity in tau-induced degeneration, we treated WT hTau^+^ neurons with pan-caspase inhibitor Z-VAD-FMK and observed a significant lessening of neurotoxicity (Figs 3G and 3H). These results suggest that caspase 3 activity contributes to WT human tau-driven neurodegeneration.

### Wild-type hTau induces DNA damage response in neurons

**Figure 4:**
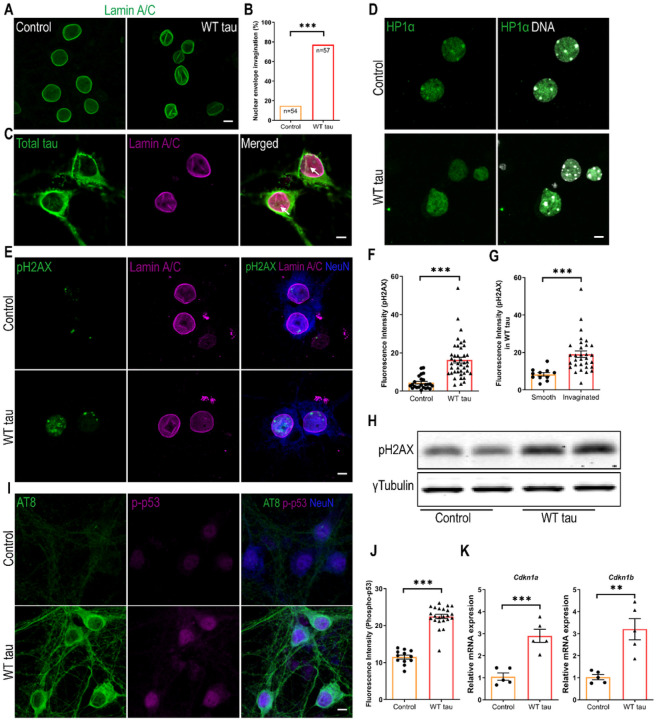
DNA damage response in WT hTau-expressing neurons. **(A)** Representative images of lamin A/C staining in control and WT tau groups at day 7 post-infection. The scale bar represents 5 μm. **(B)** Quantification of nuclear envelope invagination rate in control and WT tau groups. n=54, 57 neurons in control and WT tau groups, respectively. **(C)** Representative images of total tau and lamin A/C co-staining in WT tau group. The white arrows indicate colocalization of tau and lamin A/C in the invaginated area of nuclear membrane. The scale bar represents 5 μm. **(D)** Representative images of HP1α staining (green) in control and WT tau groups. Genomic DNA stained by DAPI is shown in white. The scale bar represents 5 μm. **(E)** Representative images of pH2AX and lamin A/C co-staining in control and WT tau groups at day 7 post-infection. The scale bar represents 5 μm. **(F)** Quantification of nuclear pH2AX signals from cultures shown in **E**. n=24, 42 neurons in control and WT tau groups, respectively. **(G)** Quantification of nuclear pH2AX signals in WT tau-expressing neurons with smooth or invaginated membrane. n=11 neurons with smooth nuclear membrane; n=31 neurons with invaginated membrane. **(H)** Western blot result of pH2AX expression in control and WT tau groups. **(I)** Representative images of AT8 and phospho-p53 (p-p53) co-staining in control and WT tau groups at day 7 post-infection. The scale bar represents 5 μm. **(J)** Quantification of nuclear p-p53 signals from cultures shown in **I**. n=12, 23 neurons in control and WT tau groups, respectively. **(K)** Quantitative RT-PCR result of relative *Cdkn1a* and *Cdkn1b* mRNA expression in control and WT tau groups at day 7 post-infection. n=5 samples/group. Data are presented as mean ± SEM. Statistical significance was determined using the Fisher’s exact test in B, or unpaired, two-tailed Student’s t-test in F, G, J and K.

To probe the mechanistic connection between tau and apoptosis, we assessed the nuclei of neurons expressing WT hTau. Lamins are architectural proteins that confer mechanical stability to the nuclear envelope [25]. Visualization of nuclear lamins revealed that, in the presence of WT hTau, many neurons displayed abnormal invaginations in their nuclear membrane (Figs 4A and 4B). Further analysis revealed the colocalization of hTau with ruffled lamin A/C signals in the nuclear envelope (Fig 4C), suggesting a physical presence of WT hTau at sites of disruption.

Nuclear envelope disturbance can have serious consequences for a cell, particularly detrimental for chromatin and genomic stability [25]. We found that WT hTau^+^ neurons displayed fewer nuclear foci of heterochromatin protein 1α (HP1α), an indication of the loss of chromatin compactness (Fig 4D). Phosphorylated Ser-139 of the histone variant H2AX (pH2AX) is a sensitive molecular marker of double-strand DNA (dsDNA) damage and repair [26]. Inside the nuclei of WT hTau^+^ neurons, we detected significantly increased pH2AX signal intensity, which correlated with the higher abundance of pH2AX protein in the cells (Figs 4E, 4F and 4H). Furthermore, we found heightened nuclear pH2AX signal in cells exhibiting nuclear membrane invagination (Fig 4G), suggesting a functional connection between nuclear envelope disruption and DNA damage response (DDR).

Activation of the well-known tumor suppressor p53 occurs in response to DNA damage and other cellular stresses and plays a critical role in apoptosis [27]. In conjunction with the pH2AX signal, phospho-p53 protein was detected in the nuclei of WT hTau^+^ neurons (Fig 4I, J). During DDR, p53 induces cell cycle regulatory proteins to elicit intrinsic checkpoint control [28]. Accordingly, we found that WT hTau^+^ neurons increased the transcription of *Cdkn1a* and *Cdkn1b*, which encode inhibitors for cyclin-dependent kinases (Fig 4K). These results collectively pinpoint a prevalent DNA damage response that is triggered by WT human tau in differentiated neurons.

### MAPK-DLK signaling partakes in WT hTau-dependent neurodegeneration

**Figure 5:**
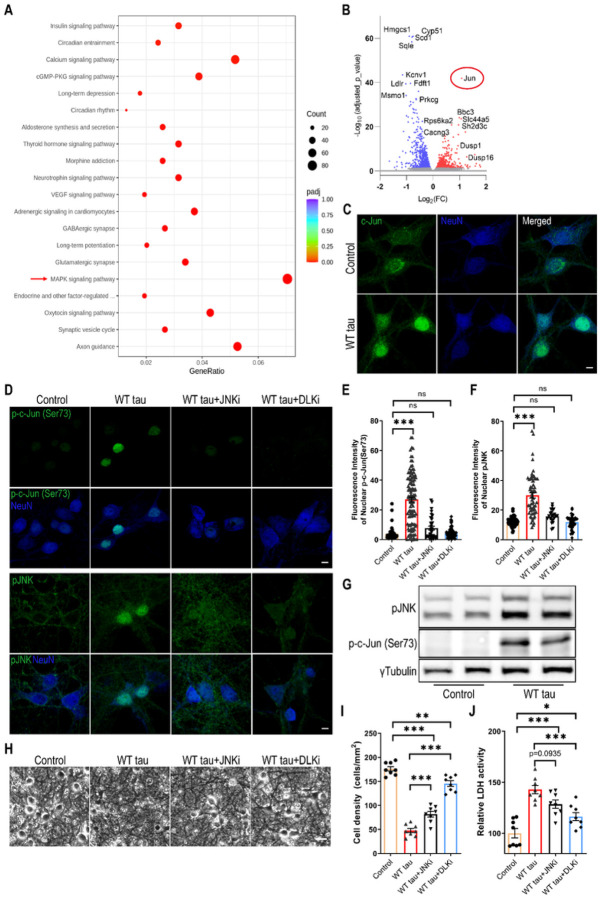
MAPK-DLK signaling in WT hTau-expressing neurons. **(A)** Top 20 most significantly affected KEGG pathways in WT tau group, compared with control group. **(B)** Volcano plot showing the top most differentially expressed genes (DEG) by WT tau. **(C)** Representative images of c-Jun staining in control and WT tau groups. The scale bar represents 5 μm. **(D)** Representative images of p-c-Jun (Ser73) and pJNK staining in control or WT tau grouped with or without JNK inhibitor (JNKi) or DLK inhibitor (DLKi) treatment. The scale bar represents 5 μm. **(E)** Quantification of nuclear pJNK signals from cultures shown in **D**. n=45, 115, 34, 90 neurons in control, WT tau, WT tau + JNKi, WT tau + DLKi groups, respectively. **(F)** Quantification of nuclear p-c-Jun (Ser73) signals from cultures shown in **D**. n=59, 62, 23, 37 neurons in control, WT tau, WT tau + JNKi, WT tau + DLKi groups, respectively. **(G)** Western blot result of pJNK and p-c-Jun (Ser73) expression in control and WT tau groups. **(H)** Representative cell images in control or WT tau groups with or without JNKi or DLKi treatment. The scale bar represents 25 μm. **(I)** Quantification of cell density from cultures shown in **H**. n=8 images/group. **(J)** Quantification of relative LDH activity in culture media of cells shown in **H**. n=8 wells/group. Data are presented as mean ± SEM. Statistical significance was determined using one-way ANOVA with Tukey’s multiple comparisons test in E, F, I and J.

To gain deeper molecular insights into the cellular pathways that are affected by WT hTau, we performed RNAseq analysis on the AAV-transduced neurons. A significant number of genes were differentially regulated between the AAV control and WT hTau^+^ neurons. Among these, multiple apoptosis and cell death-related genes displayed significantly higher levels of expression in WT hTau^+^ neurons (Fig S2A). KEGG pathway analysis further revealed multiple significantly affected cellular processes (Fig 5A; supplemental data S1). Consistent with the observed axonal and synaptic degeneration, “axon guidance” and “synaptic vesicle cycle” represented the topmost significantly altered processes. Interestingly, the “MAPK signaling pathway” contained the greatest number of genes significantly affected by WT hTau (Fig 5A). Among the genes differentially upregulated by WT hTau, *Jun* proto-oncogene ranked highest by the adjusted *p* value (Fig 5B; supplemental data S2).

c-Jun, the protein product of *Jun*, can be phosphorylated by c-Jun N-terminal kinases (JNKs), a subset of MAP kinases, and subsequently translocate to nucleus to take a part in the transcriptional activity of AP-1 [29]. Consistent with the upregulation of the *Jun* transcript, the c-Jun protein was expressed more abundantly and exhibited a nuclear enrichment in WT hTau^+^ neurons (Fig 5C). Moreover, phosphorylated c-Jun at both serine 63 and serine 73 increased in these cells, which was exclusively present inside the nuclei (Figs 5D, 5F, 5G, and S2B-C). In addition, we detected highly elevated nuclear phospho-JNK expression in many WT hTau^+^ neurons (Figs 5D, 5E, 5G). Together with the RNA profiling results, these findings uncover a substantially activated MAPK-JNK pathway in degenerating WT hTau^+^ neurons. JNK has both pro- and anti-apoptotic functions, depending on many complex factors [30]. To gauge its involvement in our model, we deployed a selective JNK inhibitor at a dose effective to diminish the activities of JNK and c-Jun in the culture of WT tau^+^ neurons (Figs 5D–5F) and observed substantial reduction of neurotoxicity with the treatment (Figs 5H–5J).

DLK is a MAP3K functionally involved in axonal degeneration as well as regeneration, generally in sync with the activities of JNKs and c-Jun [31–34]. We hypothesized that DLK activity may serve as a key node of the MAPK pathway in WT hTau^+^ neurons thus decided to examine the potency of an established DLK inhibitor to curb JNK/c-Jun signaling [35]. Similar to the JNK inhibitor, DLK inhibition effectively repressed the activities of JNK and c-Jun in hTau+ neurons (Figs 5D–5F). More potent than the JNK inhibitor, DLK inhibition significantly rescued the neurons from WT hTau-induced neurotoxicity (Fig 5H–J). Altogether, we have identified MAPK-DLK signaling as a significant contributor to WT hTau-induced neurodegeneration.

### P301L hTau overexpression differentially affects primary neurons

**Figure 6:**
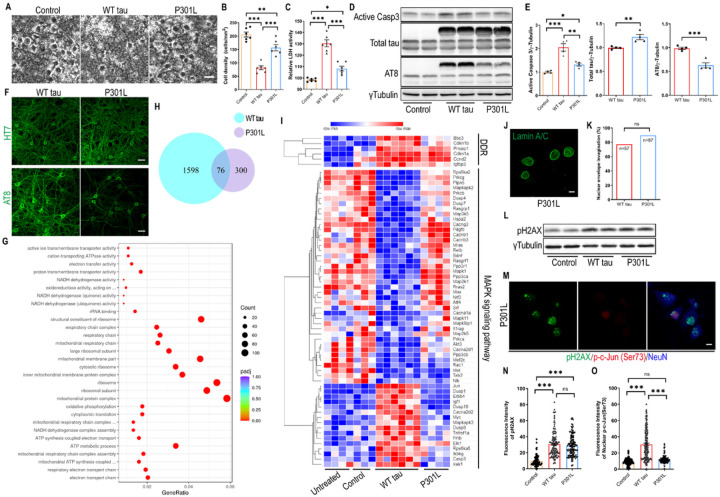
Differential effects of P301L and WT hTau on primary neurons. **(A)** Representative cell images in control, WT tau, P301L tau (P301L) groups at day 9 post-infection. Scale bar represents 25 μm. **(B)** Quantification of cell density from cultures shown in **A**. n=6 images/group. **(C)** Relative LDH activity in culture media from cultures shown in **A**. n=6 wells/group. **(D)** Western blot showing active caspase 3, total tau and AT8 expression in control, WT tau and P301L tau groups at day 7 post-infection. **(E)** Quantification of active caspase 3, exogenous total tau and AT8 expression. n=4 samples/group. **(F)** Representative images of HT7 and AT8 staining in WT tau or P301L tau groups. The scale bar represents 20 μm. **(G)** Top 30 most significantly affected pathways in P301L tau group, compared with control group. **(H)** Venn diagram of DEGs affected by WT versus P301L tau in neurons. **(I)** Heatmap showing differential expression of DNA damage response (DDR) and MAPK signal pathway genes among different groups. The rows in the heatmap were sorted by similarity via hierarchical clustering. **(J)** Representative images of lamin A/C staining in P301L group. The scale bar represents 5 μm. **(K)** Quantification of nuclear envelope invagination rate in WT tau and P301L tau groups. n=57, 87 neurons in control and WT tau groups, respectively. **(L)** Western blot result of pH2AX expression in control, WT tau and P301L tau groups at day 7 post-infection. **(M)** Representative images of pH2AX and p-c-Jun (Ser73) co-staining in P301Ltau group. The scale bar represents 5 μm. **(N)** Quantification of pH2AX signals in control, WT tau, and P301L tau groups. n=82, 124, 123 neurons in control, WT tau, P301L tau groups, respectively. **(O)** Quantification of nuclear p-c-Jun (Ser73) signals in control, WT tau, P301L tau groups. n=130, 146, 120 neurons in control, WT tau, P301L groups, respectively. Data are presented as mean ± SEM. Statistical significance was determined using unpaired, two-tailed Student’s t test in E (total tau and AT8), Fisher’s exact test in K, or one-way ANOVA with Tukey’s multiple comparisons test in B, C, E (active caspase 3), N and O.

The P301Lmissense mutation in *MAPT* is causally associated with human FTLD and has been extensively studied in various tauopathy models [12, 36]. To compare its functional impact relative to WT hTau, we similarly infected primary neurons with P301L *MAPT* packaged into an identical AAV vector to achieve equivalent overexpression. By morphological examination and measurement of LDH release, we unexpectedly observed a reduced extent of neurodegeneration by P301L hTau in side-by-side examination with WT hTau (Figs 6A–C). Consistently and significantly, P301L hTau induced less cytotoxicity than WT hTau on primary neurons in all time points examined (Fig S3A). In line with these observations, we detected lower amounts of activated caspase 3 protein in mutant hTau^+^ neurons (Figs 6D and 6E). To comprehend the underlying mechanism, we carefully compared the hTau protein expression and found that P301L hTau was somewhat less phosphorylated, even though the total abundance of transgenic tau protein was slightly higher than in cells expressing WT hTau (Figs 6D–6F, S1A). Despite a ubiquitous distribution of the mutant hTau protein, phosphorylated P301L hTau was largely restricted to somas of neurons, an intriguing contrast to phosphorylated WT hTau which abundantly associated with axons and dendrites (Fig 6F). No thioflavin S^+^ signal was detected in these cells (Fig S3B), suggesting the lack of NFT deposition, similar to WT hTau.

For a deeper understanding of changes initiated by mutated tau, we profiled the transcriptome of P301L hTau^+^ neurons and obtained a list of genes that were differentially regulated by the mutant tau. Pathway analysis revealed that P301L hTau significantly affected processes such as “electron transport chain”, “mitochondrial protein complex”, and “ribosome/ribosomal subunit” (Fig 6H; supplemental data S3). By contrast, WT hTau altered the expression of over 1600 genes in neurons, more than 3 times over mutant hTau (Fig 6G; supplemental data S2). A direct genome-wide comparison between WT and P301L hTau+ neurons uncovered selective alterations of “axon guidance” and “apoptosis” by WT hTau, and “ribosome” and “oxidative phosphorylation” by P301L hTau (Fig S3C). In terms of DNA damage response, the two hTau forms elicited similar gene expression profiles (Fig 6I); however, P301L hTau did not disrupt MAPK pathway genes as did the WT hTau (Fig 6I).

To confirm these findings, we examined P301L hTau^+^ neurons and detected many cells with invaginated nuclear envelope, similar to WT hTau^+^ neurons (Figs 6J and 6K). Likewise, increased pH2AX signal, comparable with WT hTau, was detected in P301L hTau^+^ neurons (Figs 6L–6N). By contrast, P301L hTau^+^ neurons contained much lower detectable levels of phospho-c-Jun in their nuclei in comparison with WT hTau (Figs 6M and 6O), consistent with RNAseq profiling showing a major difference in MAPK pathway between the two forms of hTau. Moreover, P301L hTau^+^ neurons showed little sign of axonal degeneration (Fig S3D). Altogether, our investigation reveals that P301L hTau overexpression differentially affected primary neurons other than WT hTau in many important aspects.

### DNA damage response and MAPK-DLK signaling synergistically promote neurodegeneration

**Figure 7:**
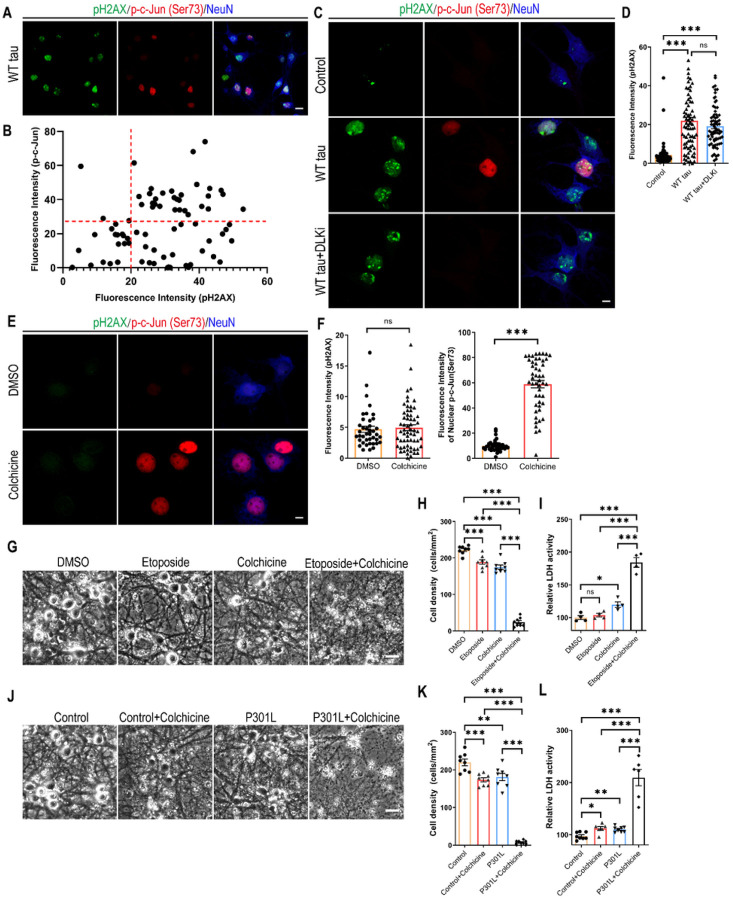
Synergy between DNA damage response and MAPK-DLK signaling in neurodegeneration. **(A)** Representative images of pH2AX and p-c-Jun (Ser73) co-staining in WT tau group. The scale bar represents 10 μm. **(B)** The correlation analysis of nuclear pH2AX and p-c-Jun (Ser73) signals in culture as shown in **A**. Total of 76 neurons were subjected to Pearson correlation analysis. R^2^=0.0492, p=0.0542. **(C)** Representative images of pH2AX and p-c-Jun (Ser73) co-staining in control and WT tau groups with or without DLK inhibitor treatment. The scale bar represents 5 μm. **(D)** Quantification of nuclear pH2AX signals in cultures as shown in **C**. n=63, 78, 72 neurons in control, WT tau, WT tau + DLKi groups, respectively. **(E)** Representative images of pH2AX and p-c-Jun (Ser73) co-staining in vehicle DMSO- and colchicine-treated groups. The scale bar represents 5 μm. **(F)** Quantification of nuclear pH2AX and p-c-Jun (Ser73) signals in cultures as shown in **C**. n=41, 50 neurons in DMSO, colchicine groups, respectively. **(G)** Representative cell images in groups treated with DMSO, etoposide, colchicine, and etoposide plus colchicine. The scale bar represents 25 μm. **(H)** Quantification of cell density in cultures as shown in **G**. n=8 images/group. **(I)** Relative LDH activity in culture media of cells as shown in **G**. n=4 wells/group. **(J)** Representative cell images in control and P301L tau groups, treated with vehicle or colchicine. The scale bar represents 25 μm. **(K)** Quantification of cell density in cultures as shown in **J**. n=8 images/group. **(L)** Relative LDH activity in culture media of cells as shown in **J**. n=8, 6, 8, 6 wells in the above groups, respectively. Data are presented as mean ± SEM. Statistical significance was determined using one-way ANOVA with Tukey’s multiple comparisons test in D, H, I, K and L, or unpaired, two-tailed Student’s t test in F.

To comprehend the relationship between DDR and MAPK-DLK activation in WT tauopathy, we took a closer look at the WT hTau^+^ neurons by dual immunostaining of pH2AX and phospho-c-Jun and detected more cells expressing the marker of DDR than those with MAPK signaling (Fig 7A). We did not find a significant correlation between the levels of the two markers on a per cell basis, but noticed a general lack of cells solely expressing nuclear phospho-c-Jun (Figs 7A and 7B). Interestingly, WT hTau^+^ neurons treated with DLK inhibitor did not diminish the pH2AX signal (Figs 7C and 7D), suggesting that DNA damage response is either upstream or independent of MAPK-DLK signaling.

Pathogenic tau is known to modify cytoskeletal functions by dissociating from microtubules and intercepting F-actin [37, 38]. Having observed distinct distribution patterns of WT and P301L phospho-hTau (Fig 6F) and selective accumulation of activated caspase 3 in axonal spheroids in WT hTau^+^ neurons (Fig 2A), we hypothesized that 1) axonal WT hTau may stimulate MAPK-DLK signaling by inducing axonal degeneration independently from somatic hTau-induced DDR; and 2) MAPK-DLK signaling and DDR function synergistically in WT hTau^+^ neurons, whereas lack of axonal DLK activation renders P301L tau less neurotoxic. To test this hypothesis, we adopted a selective inhibitor of microtubule formation [39], as disruption of microtubule dynamics is known to cause DLK-mediated axonal swelling and degeneration [40]. Low-dose colchicine treatment robustly induced nuclear phospho-c-Jun accumulation without any sign of DDR in primary neurons (Figs 7E and 7F). We also used etoposide, a chemotherapy agent capable of inducing dsDNA breaks: primary neurons exposed to low-dose etoposide readily upregulated nuclear pH2AX expression (Fig S4). Individually, these compounds caused limited toxicity in neurons after 48 hours; however, combined treatment resulted in severe cell loss and LDH release, indicating a powerful synergistic effect of axonal stress and DDR in neurodegeneration (Figs 7G–7I). To further demonstrate the interaction between these pathways in the tau-relevant situation, we treated P301L hTau^+^ neurons with low-dose colchicine and observed significantly expedited degeneration in culture (Figs 7J–7L). In summary, these findings uncover a striking synergistic interplay between DNA damage response and the MAPK-DLK axis in AD-relevant neurodegeneration.

## Discussion

In this study, we investigated the consequence of human tau overexpression in primary neurons and discovered a pathogenic coupling of DNA damage response and MAPK-DLK signaling in wild-type hTau-mediated neurotoxicity. By contrast, P301L hTau, which is associated with FTLD, elicited primarily the DNA damage response, with less aggressive neurodegeneration. The overt WT hTau-induced pathology was highly significant and reproducible, yet somewhat unexpected. Historically, non-mutant *MAPT*-expressing mouse strains either develop no CNS NFT or limited tangle pathology only when the animals are aged [12, 36]. Instead, overexpression of mutant *MAPT* genes results in robust NFT deposition in rodent brains, which led to the wide adaptation of the mutant models to study tauopathy. It becomes clear now that transgene expression *in vivo* is grossly affected by the promoter of choice as well as transgene copy number and insertion sites, making it difficult to directly compare different transgenic lines. Interestingly, Gamache et al constructed genetically matched transgenic mice overexpressing WT or P301L 0N4R hTau and observed greater pathogenicity in WT hTau^+^ animals, including exaggerated tau hyperphosphorylation and early cognitive impairment [41]. Although we studied the 2N4R isoform *in vitro*, the phenotypic disparity between WT and mutant hTau is highly analogous.

AD and other tauopathies may affect different brain regions, thus their pathogenesis can be mediated by different types of neurons, circuits, and other cell types under the influence of distinct local signals. Nevertheless, our cortical neuron culture revealed a remarkable contrast between WT and mutant hTau in altering key cellular processes, highlighting a keen influence by the identity of the tau protein itself in disease progression. Extensive biochemical and biophysical characterization has revealed structural distinctions between NFTs formed under different tauopathy conditions, of which sensitive tracers can be applied to detect their deposition by positron emission tomography [42, 43]. In addition to altered binding to microtubules, WT hTau interacts with a long list of cellular proteins not observed with FTLD-associated mutants [16, 38, 44, 45]. For example, Tracy *et al* performed an in-depth analysis of the tau interactome in human iPSCs and reported preferential interaction of WT tau with mitochondria and impaired bioenergetics by FTLD-tau [16]. Not so surprisingly, WT and mutant hTau also differentially affect the global transcriptome. Using tau-inducible HEK cells, Montalbano *et al* reported that WT hTau affected a higher number of genes than P301L mutant, including those involved in cytoskeleton-dependent processes, while P301L hTau perturbed pathways associated with reactive oxygen species [17]. In fully differentiated neurons, we similarly observed more profound influence of WT hTau on transcriptome (Fig. 6H) and detected selective effects on “axon guidance” and “apoptosis” by WT hTau, and “ribosome” and “oxidative phosphorylation” by P301L hTau (Fig S3).

AD and tauopathies are associated with numerous nuclear irregularities, which include excessive DNA damage [46, 47], altered DNA repair [21, 48], cell cycle re-entry [19, 49], chromosomal defects [50] and senescence [51, 52]. Long linked to brain aging and neurodegeneration, neuronal DNA damage, manifested by pH2AX^+^ foci, accumulates early in AD brain [51, 52]. Here, we detected comparable levels of pH2AX signal associated with WT and mutant hTau in primary neurons. Very low levels of tau localizes inside the nuclei of healthy neurons, where it plays a role in regulating genome stability and nucleolar function [53]. Although we did not observe increased nuclear tau, our characterization revealed close proximity of hTau with the nuclear membrane, which is accompanied by significant nuclear envelope deformation (Figs. 4 and 6). This finding is consistent with earlier reports on human AD as well as the aberrant interaction between pathogenic tau and nuclear pore components [54–58]. Nuclear envelope disruption may also impair the structures that anchor heterochromatin and cause genomic injury [59]. Our examination revealed abnormal heterochromatin relaxation and dsDNA damage response in hTau^+^ neurons (Fig. 4). While tau-associated global chromatin relaxation has been reported in human AD and identified as a toxic effector of neurodegeneration in a tauopathy model, tau accumulation was also shown to trigger DDR by various studies [21, 58, 60, 61]. DNA damage is a well-known stress inducer of apoptosis [62]. In WT hTau^+^ neurons, we observed an enrichment of the “apoptosis” pathway, the upregulation of several prototypical executors of apoptosis, such as caspase-3 and PUMA, and detected the functional involvement of caspase activation (Figs. 3, S2 and S3).

Neurons are highly sensitive to stress, which can activate multiple signaling pathways and eventually lead to cell death governed by a variety of mechanisms [63, 64]. Axonal degeneration is of particular importance, as axonal cytoskeleton integrity is essential for many neuronal functions, such as long-range cargo transport and transmission of action potentials [65]. We have detected several hallmarks of axonal degeneration in WT hTau^+^ neurons: prominent formation of axonal spheroids colocalized with activated caspase 3 and accumulation of fragmented neurofilaments, accompanied by the loss of synapses and eventual release of LDH and cell death (Figs. 2 and 3). In contrast, little axonal degeneration was detectable in P301L hTau^+^ neurons (Fig S3D). Coincidently, we found that WT hTau, hyperphosphorylated and aggregated, is abundantly distributed throughout the axons and dendrites. This is in sharp contrast with P301L hTau, which was largely restricted to the soma of neurons when phosphorylated (Figs. 2 and 6). Consistent with our *in vitro* findings, axonopathy represents the most robust pathological feature exhibited by various WT hTau animal models *in vivo*, which was remarkably absent in mutant *MAPT*-expressing brains [66–69].

The activity of DLK, a neuron-specific MAP3K, is centrally involved in both axonal degeneration and regeneration, and its pathogenic role has been implicated in neurodegeneration [35, 70]. Intriguingly, DLK’s downstream target JNK and its substrate c-Jun were increased in WT hTau^+^ neurons, mirrored by the enrichment of the MAPK pathway in these cells (Fig. 5). Furthermore, we showed that pharmacological inhibition of DLK was highly effective in rescuing WT hTau^+^ neurons, implying a prominent role played by the MAPK-DLK axis in AD-relevant neurodegeneration (Fig. 5). Axonopathy and transport deficits occur early in the pathogenesis of AD when axonal swellings are abundant near the amyloid plaques [71]. Not merely appearing in Braak I-III, phospho-tau^+^ axonal spheroids continuously and robustly build up in later stages (Braak IV-VI) of the disease [71–73]. Remarkably, expression of phospho-JNK and phospho-c-Jun correlates positively with phosphorylated pathogenic tau in AD brains [35]. Notably, the MAPK pathway has been independently identified by recent proteomic studies as one of the most significantly affected processes in AD [74, 75]. Hence, our findings here offer mechanistic insight into how the DLK-JNK-c-Jun axis, one discrete branch of the MAPK cascade, directly partakes in neuron-intrinsic degeneration in AD.

We have focused on investigating the neuronal intrinsic effects of human tau in this study. As a result, the major limitation is the absence of glial cells in the model. We aim to develop *in vivo* tauopathy models using the same set of AAV vectors to dissect the interaction between hTau^+^ neurons and different glial populations that play critical roles in neuronal support as well as pathogenesis [81, 82]. We have only characterized the 2N4R form so far, but both 3R and 4R tau isoforms are implicated in AD. Since the hTau protein is expressed in the presence of endogenous mouse tau, we cannot rule out a potential interference by mouse tau. Therefore, we plan to elucidate the impacts of WT hTau isoforms and mutant forms in differentiated human neurons in the future.

## Conclusions

Tau-mediated pathogenesis of CNS diseases is exceedingly complex in terms of its underlying mechanism, which may involve losses and/or gains of function. Pathogenic tau species formed *de novo* in disease are believed to play a major role in tauopathies. Despite being the disease’s pathological hallmark, insoluble NFT *per se* may not necessarily be neurotoxic [1, 2]. Multiple studies have highlighted soluble oligomeric assemblies or fragments of tau protein as the more harmful forms of abnormal tau [76–79]. In our cellular tauopathy model, hyperphosphorylated and aggregated full-length hTau was sufficient to induce neurotoxicity in the absence of NFT formation. Remarkably in WT hTau^+^ neurons, soluble pathogenic tau simultaneously initiates two major pathways from distinct compartments: DNA damage response in the perinuclear area, and MAPK-DLK activation in distal axons. We have demonstrated that these two pathways synergistically promote cytotoxicity in neurons, an exciting discovery with both scientific and therapeutic implications. Stress signaling has been shown to couple with DLK to drive degenerative process [80]. Here our findings identify a functional coupling of DNA damage response, a major cellular stressor, with DLK activity in AD-relevant tauopathy. The vital pathogenic involvement of MAPK-DLK axis solidifies DLK as a potential therapeutic target, especially in the later stages of the disease, and provides a rationale to explore DLK inhibitors in treating AD.

## Data Availability

In addition to results included in supplementary data 1–4, RNAseq data will be deposited at GEO and publicly available as of the date of publication. Any additional information related to the data reported in this paper is available from the corresponding author upon reasonable request.

## Abbreviations

AAV: Adeno-associated virus
AD: Alzheimer’s disease
AP-1: Activator protein 1
CBD: Corticobasal degeneration
CNS: Central nervous system
DDR: DNA damage response
DLK: Dual leucine zipper kinase
DLKi: DLK inhibitor
dsDNA: Double stranded DNA
FTLD: Frontotemporal lobar degeneration
hTau: Human tau
JNK: c-Jun N-terminal kinase
JNKi: JNK inhibitor
LDH: Lactate dehydrogenase
MAP3K: MAP kinase kinase kinase
MAPK: Mitogen-activated protein kinase
MAPT: Microtubule associated protein tau
NFT: Neurofibrillary tangles
p-c-Jun: Phospho-c-Jun
pH2AX: Phospho-Histone H2A.X
p-JNK: Phospho-JNK
pNF-H: Phospho-Neurofilament H
p-p53: Phospho-p53
PSP: Progressive supranuclear palsy
RNAseq: RNA Sequencing
SYN1: Synapsin I
ThioS: Thioflavin S
WT: Wild-type
Z-VAD: Z-VAD-FMK

## References

[1] Chang CW, Shao E, Mucke L: Tau: Enabler of diverse brain disorders and target of rapidly evolving therapeutic strategies. Science 2021, 371. 10.1126/science.abb8255 PMC8118650 33632820

[2] Wang Y, Mandelkow E: Tau in physiology and pathology. Nat Rev Neurosci 2016, 17:5–21. 26631930 10.1038/nrn.2015.1

[3] Tracy TE, Gan L: Tau-mediated synaptic and neuronal dysfunction in neurodegenerative disease. Curr Opin Neurobiol 2018, 51:134–138. 29753269 10.1016/j.conb.2018.04.027 PMC6130905

[4] Guo T, Noble W, Hanger DP: Roles of tau protein in health and disease. Acta Neuropathol 2017, 133:665–704. 28386764 10.1007/s00401-017-1707-9 PMC5390006

[5] Goedert M, Jakes R: Mutations causing neurodegenerative tauopathies. Biochim Biophys Acta 2005, 1739:240–250. 15615642 10.1016/j.bbadis.2004.08.007

[6] Dujardin S, Commins C, Lathuiliere A, Beerepoot P, Fernandes AR, Kamath TV, De Los Santos MB, Klickstein N, Corjuc DL, Corjuc BT, : Tau molecular diversity contributes to clinical heterogeneity in Alzheimer’s disease. Nat Med 2020, 26:1256–1263. 32572268 10.1038/s41591-020-0938-9 PMC7603860

[7] Wesseling H, Mair W, Kumar M, Schlaffner CN, Tang S, Beerepoot P, Fatou B, Guise AJ, Cheng L, Takeda S, : Tau PTM Profiles Identify Patient Heterogeneity and Stages of Alzheimer’s Disease. Cell 2020, 183:1699–1713 e1613. 33188775 10.1016/j.cell.2020.10.029 PMC8168922

[8] Knopman DS, Amieva H, Petersen RC, Chetelat G, Holtzman DM, Hyman BT, Nixon RA, Jones DT: Alzheimer disease. Nat Rev Dis Primers 2021, 7:33. 33986301 10.1038/s41572-021-00269-y PMC8574196

[9] Scheltens P, De Strooper B, Kivipelto M, Holstege H, Chetelat G, Teunissen CE, Cummings J, van der Flier WM: Alzheimer’s disease. Lancet 2021, 397:1577–1590. 33667416 10.1016/S0140-6736(20)32205-4 PMC8354300

[10] Verheyen A, Diels A, Dijkmans J, Oyelami T, Meneghello G, Mertens L, Versweyveld S, Borgers M, Buist A, Peeters P, Cik M: Using Human iPSC-Derived Neurons to Model TAU Aggregation. PLoS One 2015, 10:e0146127. 26720731 10.1371/journal.pone.0146127 PMC4697850

[11] Koller EJ, Gonzalez De La Cruz E, Machula T, Ibanez KR, Lin WL, Williams T, Riffe CJ, Ryu D, Strang KH, Liu X, : Combining P301L and S320F tau variants produces a novel accelerated model of tauopathy. Hum Mol Genet 2019, 28:3255–3269. 31261380 10.1093/hmg/ddz151 PMC6859436

[12] Gotz J, Streffer JR, David D, Schild A, Hoerndli F, Pennanen L, Kurosinski P, Chen F: Transgenic animal models of Alzheimer’s disease and related disorders: histopathology, behavior and therapy. Mol Psychiatry 2004, 9:664–683. 15052274 10.1038/sj.mp.4001508

[13] Bucci M, Chiotis K, Nordberg A, Alzheimer’s Disease Neuroimaging I: Alzheimer’s disease profiled by fluid and imaging markers: tau PET best predicts cognitive decline. Mol Psychiatry 2021, 26:5888–5898. 34593971 10.1038/s41380-021-01263-2 PMC8758489

[14] Aschenbrenner AJ, Gordon BA, Benzinger TLS, Morris JC, Hassenstab JJ: Influence of tau PET, amyloid PET, and hippocampal volume on cognition in Alzheimer disease. Neurology 2018, 91:e859–e866. 30068637 10.1212/WNL.0000000000006075 PMC6133625

[15] Jankowsky JL, Zheng H: Practical considerations for choosing a mouse model of Alzheimer’s disease. Molecular neurodegeneration 2017, 12:89. 29273078 10.1186/s13024-017-0231-7 PMC5741956

[16] Tracy TE, Madero-Perez J, Swaney DL, Chang TS, Moritz M, Konrad C, Ward ME, Stevenson E, Huttenhain R, Kauwe G, : Tau interactome maps synaptic and mitochondrial processes associated with neurodegeneration. Cell 2022, 185:712–728 e714. 35063084 10.1016/j.cell.2021.12.041 PMC8857049

[17] Montalbano M, Jaworski E, Garcia S, Ellsworth A, McAllen S, Routh A, Kayed R: Tau Modulates mRNA Transcription, Alternative Polyadenylation Profiles of hnRNPs, Chromatin Remodeling and Spliceosome Complexes. Front Mol Neurosci 2021, 14:742790. 34924950 10.3389/fnmol.2021.742790 PMC8678415

[18] Holmes BB, Furman JL, Mahan TE, Yamasaki TR, Mirbaha H, Eades WC, Belaygorod L, Cairns NJ, Holtzman DM, Diamond MI: Proteopathic tau seeding predicts tauopathy in vivo. Proc Natl Acad Sci U S A 2014, 111:E4376–4385. 25261551 10.1073/pnas.1411649111 PMC4205609

[19] Jaworski T, Dewachter I, Lechat B, Croes S, Termont A, Demedts D, Borghgraef P, Devijver H, Filipkowski RK, Kaczmarek L, : AAV-tau mediates pyramidal neurodegeneration by cell-cycle re-entry without neurofibrillary tangle formation in wild-type mice. PLoS One 2009, 4:e7280. 19794916 10.1371/journal.pone.0007280 PMC2748684

[20] Roy ER, Chiu G, Li S, Propson NE, Kanchi R, Wang B, Coarfa C, Zheng H, Cao W: Concerted type I interferon signaling in microglia and neural cells promotes memory impairment associated with amyloid beta plaques. Immunity 2022, 55:879–894 e876. 35443157 10.1016/j.immuni.2022.03.018 PMC9109419

[21] Farmer KM, Ghag G, Puangmalai N, Montalbano M, Bhatt N, Kayed R: P53 aggregation, interactions with tau, and impaired DNA damage response in Alzheimer’s disease. Acta neuropathologica communications 2020, 8:132. 32778161 10.1186/s40478-020-01012-6 PMC7418370

[22] Behl C: Apoptosis and Alzheimer’s disease. J Neural Transm (Vienna) 2000, 107:1325–1344. 11145007 10.1007/s007020070021

[23] Porter AG, Janicke RU: Emerging roles of caspase-3 in apoptosis. Cell Death Differ 1999, 6:99–104. 10200555 10.1038/sj.cdd.4400476

[24] Nakano K, Vousden KH: PUMA, a novel proapoptotic gene, is induced by p53. Mol Cell 2001, 7:683–694. 11463392 10.1016/s1097-2765(01)00214-3

[25] Mekhail K, Moazed D: The nuclear envelope in genome organization, expression and stability. Nat Rev Mol Cell Biol 2010, 11:317–328. 20414256 10.1038/nrm2894 PMC3246372

[26] Mah LJ, El-Osta A, Karagiannis TC: gammaH2AX: a sensitive molecular marker of DNA damage and repair. Leukemia 2010, 24:679–686. 20130602 10.1038/leu.2010.6

[27] Fridman JS, Lowe SW: Control of apoptosis by p53. Oncogene 2003, 22:9030–9040. 14663481 10.1038/sj.onc.1207116

[28] Zhou BB, Elledge SJ: The DNA damage response: putting checkpoints in perspective. Nature 2000, 408:433–439. 11100718 10.1038/35044005

[29] Bohmann D, Bos TJ, Admon A, Nishimura T, Vogt PK, Tjian R: Human proto-oncogene c-jun encodes a DNA binding protein with structural and functional properties of transcription factor AP-1. Science 1987, 238:1386–1392. 2825349 10.1126/science.2825349

[30] Yarza R, Vela S, Solas M, Ramirez MJ: c-Jun N-terminal Kinase (JNK) Signaling as a Therapeutic Target for Alzheimer’s Disease. Front Pharmacol 2015, 6:321. 26793112 10.3389/fphar.2015.00321 PMC4709475

[31] Watkins TA, Wang B, Huntwork-Rodriguez S, Yang J, Jiang Z, Eastham-Anderson J, Modrusan Z, Kaminker JS, Tessier-Lavigne M, Lewcock JW: DLK initiates a transcriptional program that couples apoptotic and regenerative responses to axonal injury. Proc Natl Acad Sci U S A 2013, 110:4039–4044. 23431164 10.1073/pnas.1211074110 PMC3593899

[32] Welsbie DS, Ziogas NK, Xu L, Kim BJ, Ge Y, Patel AK, Ryu J, Lehar M, Alexandris AS, Stewart N, : Targeted disruption of dual leucine zipper kinase and leucine zipper kinase promotes neuronal survival in a model of diffuse traumatic brain injury. Molecular neurodegeneration 2019, 14:44. 31775817 10.1186/s13024-019-0345-1 PMC6882250

[33] Welsbie DS, Yang Z, Ge Y, Mitchell KL, Zhou X, Martin SE, Berlinicke CA, Hackler L Jr., Fuller J, Fu J, : Functional genomic screening identifies dual leucine zipper kinase as a key mediator of retinal ganglion cell death. Proc Natl Acad Sci U S A 2013, 110:4045–4050. 23431148 10.1073/pnas.1211284110 PMC3593842

[34] Gerdts J, Summers DW, Milbrandt J, DiAntonio A: Axon Self-Destruction: New Links among SARM1, MAPKs, and NAD+ Metabolism. Neuron 2016, 89:449–460. 26844829 10.1016/j.neuron.2015.12.023 PMC4742785

[35] Le Pichon CE, Meilandt WJ, Dominguez S, Solanoy H, Lin H, Ngu H, Gogineni A, Sengupta Ghosh A, Jiang Z, Lee SH, : Loss of dual leucine zipper kinase signaling is protective in animal models of neurodegenerative disease. Sci Transl Med 2017, 9:eaag0394. 28814543 10.1126/scitranslmed.aag0394

[36] Gotz J: Tau and transgenic animal models. Brain Res Brain Res Rev 2001, 35:266–286. 11423157 10.1016/s0165-0173(01)00055-8

[37] Fulga TA, Elson-Schwab I, Khurana V, Steinhilb ML, Spires TL, Hyman BT, Feany MB: Abnormal bundling and accumulation of F-actin mediates tau-induced neuronal degeneration in vivo. Nat Cell Biol 2007, 9:139–148. 17187063 10.1038/ncb1528

[38] Hong M, Zhukareva V, Vogelsberg-Ragaglia V, Wszolek Z, Reed L, Miller BI, Geschwind DH, Bird TD, McKeel D, Goate A, : Mutation-specific functional impairments in distinct tau isoforms of hereditary FTDP-17. Science 1998, 282:1914–1917. 9836646 10.1126/science.282.5395.1914

[39] Margolis RL, Wilson L: Addition of colchicine--tubulin complex to microtubule ends: the mechanism of substoichiometric colchicine poisoning. Proc Natl Acad Sci U S A 1977, 74:3466–3470. 269406 10.1073/pnas.74.8.3466 PMC431611

[40] Valakh V, Frey E, Babetto E, Walker LJ, DiAntonio A: Cytoskeletal disruption activates the DLK/JNK pathway, which promotes axonal regeneration and mimics a preconditioning injury. Neurobiol Dis 2015, 77:13–25. 25726747 10.1016/j.nbd.2015.02.014 PMC4402261

[41] Gamache JE, Kemper L, Steuer E, Leinonen-Wright K, Choquette JM, Hlynialuk C, Benzow K, Vossel KA, Xia W, Koob MD, Ashe KH: Developmental Pathogenicity of 4-Repeat Human Tau Is Lost with the P301L Mutation in Genetically Matched Tau-Transgenic Mice. J Neurosci 2020, 40:220–236. 31685653 10.1523/JNEUROSCI.1256-19.2019 PMC6939485

[42] Shi Y, Zhang W, Yang Y, Murzin AG, Falcon B, Kotecha A, van Beers M, Tarutani A, Kametani F, Garringer HJ, : Structure-based classification of tauopathies. Nature 2021, 598:359–363. 34588692 10.1038/s41586-021-03911-7 PMC7611841

[43] Saint-Aubert L, Lemoine L, Chiotis K, Leuzy A, Rodriguez-Vieitez E, Nordberg A: Tau PET imaging: present and future directions. Molecular neurodegeneration 2017, 12:19. 28219440 10.1186/s13024-017-0162-3 PMC5319037

[44] Wang X, Williams D, Muller I, Lemieux M, Dukart R, Maia IBL, Wang H, Woerman AL, Schmitt-Ulms G: Tau interactome analyses in CRISPR-Cas9 engineered neuronal cells reveal ATPase-dependent binding of wild-type but not P301L Tau to non-muscle myosins. Scientific reports 2019, 9:16238. 31700063 10.1038/s41598-019-52543-5 PMC6838314

[45] Bhaskar K, Yen SH, Lee G: Disease-related modifications in tau affect the interaction between Fyn and Tau. J Biol Chem 2005, 280:35119–35125. 16115884 10.1074/jbc.M505895200

[46] Coppede F, Migliore L: DNA damage in neurodegenerative diseases. Mutat Res 2015, 776:84–97. 26255941 10.1016/j.mrfmmm.2014.11.010

[47] Stein D, Toiber D: DNA damage and neurodegeneration: the unusual suspect. Neural regeneration research 2017, 12:1441–1442. 29089988 10.4103/1673-5374.215254 PMC5649463

[48] Jeppesen DK, Bohr VA, Stevnsner T: DNA repair deficiency in neurodegeneration. Prog Neurobiol 2011, 94:166–200. 21550379 10.1016/j.pneurobio.2011.04.013 PMC3123739

[49] Andorfer C, Acker CM, Kress Y, Hof PR, Duff K, Davies P: Cell-cycle reentry and cell death in transgenic mice expressing nonmutant human tau isoforms. J Neurosci 2005, 25:5446–5454. 15930395 10.1523/JNEUROSCI.4637-04.2005 PMC6725006

[50] Rossi G, Dalpra L, Crosti F, Lissoni S, Sciacca FL, Catania M, Di Fede G, Mangieri M, Giaccone G, Croci D, Tagliavini F: A new function of microtubule-associated protein tau: involvement in chromosome stability. Cell cycle 2008, 7:1788–1794. 18583940 10.4161/cc.7.12.6012

[51] Shanbhag NM, Evans MD, Mao W, Nana AL, Seeley WW, Adame A, Rissman RA, Masliah E, Mucke L: Early neuronal accumulation of DNA double strand breaks in Alzheimer’s disease. Acta neuropathologica communications 2019, 7:77. 31101070 10.1186/s40478-019-0723-5 PMC6524256

[52] Simpson JE, Ince PG, Matthews FE, Shaw PJ, Heath PR, Brayne C, Garwood C, Higginbottom A, Wharton SB, Function MRCC, Ageing Neuropathology Study G: A neuronal DNA damage response is detected at the earliest stages of Alzheimer’s neuropathology and correlates with cognitive impairment in the Medical Research Council’s Cognitive Function and Ageing Study ageing brain cohort. Neuropathol Appl Neurobiol 2015, 41:483–496. 25443110 10.1111/nan.12202 PMC4861215

[53] Bukar Maina M, Al-Hilaly YK, Serpell LC: Nuclear Tau and Its Potential Role in Alzheimer’s Disease. Biomolecules 2016, 6:9. 26751496 10.3390/biom6010009 PMC4808803

[54] Coyne AN, Rothstein JD: Nuclear pore complexes - a doorway to neural injury in neurodegeneration. Nature reviews Neurology 2022, 18:348–362. 35488039 10.1038/s41582-022-00653-6 PMC10015220

[55] Prissette M, Fury W, Koss M, Racioppi C, Fedorova D, Dragileva E, Clarke G, Pohl T, Dugan J, Ahrens D, : Disruption of nuclear envelope integrity as a possible initiating event in tauopathies. Cell Rep 2022, 40:111249. 36001963 10.1016/j.celrep.2022.111249

[56] Eftekharzadeh B, Daigle JG, Kapinos LE, Coyne A, Schiantarelli J, Carlomagno Y, Cook C, Miller SJ, Dujardin S, Amaral AS, : Tau Protein Disrupts Nucleocytoplasmic Transport in Alzheimer’s Disease. Neuron 2018, 99:925–940 e927. 30189209 10.1016/j.neuron.2018.07.039 PMC6240334

[57] Frost B, Bardai FH, Feany MB: Lamin Dysfunction Mediates Neurodegeneration in Tauopathies. Curr Biol 2016, 26:129–136. 26725200 10.1016/j.cub.2015.11.039 PMC4713335

[58] Frost B, Hemberg M, Lewis J, Feany MB: Tau promotes neurodegeneration through global chromatin relaxation. Nat Neurosci 2014, 17:357–366. 24464041 10.1038/nn.3639 PMC4012297

[59] Gonzalo S: DNA Damage and Lamins. In Cancer Biology and the Nuclear Envelope: Recent Advances May Elucidate Past Paradoxes. Edited by Schirmer EC, de las Heras JI. New York, NY: Springer New York; 2014: 377–399

[60] Asada-Utsugi M, Uemura K, Ayaki T, M TU, Minamiyama S, Hikiami R, Morimura T, Shodai A, Ueki T, Takahashi R, : Failure of DNA double-strand break repair by tau mediates Alzheimer’s disease pathology in vitro. Commun Biol 2022, 5:358. 35418705 10.1038/s42003-022-03312-0 PMC9008043

[61] Cimini S, Giaccone G, Tagliavini F, Costantino M, Perego P, Rossi G: P301L tau mutation leads to alterations of cell cycle, DNA damage response and apoptosis: Evidence for a role of tau in cancer. Biochem Pharmacol 2022, 200:115043. 35436464 10.1016/j.bcp.2022.115043

[62] Norbury CJ, Zhivotovsky B: DNA damage-induced apoptosis. Oncogene 2004, 23:2797–2808. 15077143 10.1038/sj.onc.1207532

[63] Fricker M, Tolkovsky AM, Borutaite V, Coleman M, Brown GC: Neuronal Cell Death. Physiol Rev 2018, 98:813–880. 29488822 10.1152/physrev.00011.2017 PMC5966715

[64] Farley MM, Watkins TA: Intrinsic Neuronal Stress Response Pathways in Injury and Disease. Annu Rev Pathol 2018, 13:93–116. 29414247 10.1146/annurev-pathol-012414-040354

[65] Coleman M: Axon degeneration mechanisms: commonality amid diversity. Nat Rev Neurosci 2005, 6:889–898. 16224497 10.1038/nrn1788

[66] Ishihara T, Hong M, Zhang B, Nakagawa Y, Lee MK, Trojanowski JQ, Lee VM: Age-dependent emergence and progression of a tauopathy in transgenic mice overexpressing the shortest human tau isoform. Neuron 1999, 24:751–762. 10595524 10.1016/s0896-6273(00)81127-7

[67] Spittaels K, Van den Haute C, Van Dorpe J, Bruynseels K, Vandezande K, Laenen I, Geerts H, Mercken M, Sciot R, Van Lommel A, : Prominent axonopathy in the brain and spinal cord of transgenic mice overexpressing four-repeat human tau protein. Am J Pathol 1999, 155:2153–2165. 10595944 10.1016/S0002-9440(10)65533-2 PMC1866931

[68] Terwel D, Lasrado R, Snauwaert J, Vandeweert E, Van Haesendonck C, Borghgraef P, Van Leuven F: Changed conformation of mutant Tau-P301L underlies the moribund tauopathy, absent in progressive, nonlethal axonopathy of Tau-4R/2N transgenic mice. J Biol Chem 2005, 280:3963–3973. 15509565 10.1074/jbc.M409876200

[69] Probst A, Gotz J, Wiederhold KH, Tolnay M, Mistl C, Jaton AL, Hong M, Ishihara T, Lee VM, Trojanowski JQ, : Axonopathy and amyotrophy in mice transgenic for human four-repeat tau protein. Acta Neuropathol 2000, 99:469–481. 10805089 10.1007/s004010051148

[70] Ghosh AS, Wang B, Pozniak CD, Chen M, Watts RJ, Lewcock JW: DLK induces developmental neuronal degeneration via selective regulation of proapoptotic JNK activity. J Cell Biol 2011, 194:751–764. 21893599 10.1083/jcb.201103153 PMC3171129

[71] Stokin GB, Lillo C, Falzone TL, Brusch RG, Rockenstein E, Mount SL, Raman R, Davies P, Masliah E, Williams DS, Goldstein LS: Axonopathy and transport deficits early in the pathogenesis of Alzheimer’s disease. Science 2005, 307:1282–1288. 15731448 10.1126/science.1105681

[72] Su JH, Cummings BJ, Cotman CW: Identification and distribution of axonal dystrophic neurites in Alzheimer’s disease. Brain Res 1993, 625:228–237. 8275305 10.1016/0006-8993(93)91063-x

[73] Gibson PH: Ultrastructural abnormalities in the cerebral neocortex and hippocampus associated with Alzheimer’s disease and aging. Acta Neuropathol 1987, 73:86–91. 3604576 10.1007/BF00695506

[74] Johnson ECB, Carter EK, Dammer EB, Duong DM, Gerasimov ES, Liu Y, Liu J, Betarbet R, Ping L, Yin L, : Large-scale deep multi-layer analysis of Alzheimer’s disease brain reveals strong proteomic disease-related changes not observed at the RNA level. Nat Neurosci 2022, 25:213–225. 35115731 10.1038/s41593-021-00999-y PMC8825285

[75] Swarup V, Chang TS, Duong DM, Dammer EB, Dai J, Lah JJ, Johnson ECB, Seyfried NT, Levey AI, Geschwind DH: Identification of Conserved Proteomic Networks in Neurodegenerative Dementia. Cell Rep 2020, 31:107807. 32579933 10.1016/j.celrep.2020.107807 PMC8221021

[76] Ash PEA, Lei S, Shattuck J, Boudeau S, Carlomagno Y, Medalla M, Mashimo BL, Socorro G, Al-Mohanna LFA, Jiang L, : TIA1 potentiates tau phase separation and promotes generation of toxic oligomeric tau. Proc Natl Acad Sci U S A 2021, 118:e2014188118. 10.1073/pnas.2014188118 PMC7936275 33619090

[77] Lasagna-Reeves CA, Castillo-Carranza DL, Sengupta U, Sarmiento J, Troncoso J, Jackson GR, Kayed R: Identification of oligomers at early stages of tau aggregation in Alzheimer’s disease. FASEB J 2012, 26:1946–1959. 22253473 10.1096/fj.11-199851 PMC4046102

[78] Zhang Z, Song M, Liu X, Kang SS, Kwon IS, Duong DM, Seyfried NT, Hu WT, Liu Z, Wang JZ, : Cleavage of tau by asparagine endopeptidase mediates the neurofibrillary pathology in Alzheimer’s disease. Nat Med 2014, 20:1254–1262. 25326800 10.1038/nm.3700 PMC4224595

[79] Zhao X, Kotilinek LA, Smith B, Hlynialuk C, Zahs K, Ramsden M, Cleary J, Ashe KH: Caspase-2 cleavage of tau reversibly impairs memory. Nat Med 2016, 22:1268–1276. 27723722 10.1038/nm.4199

[80] Larhammar M, Huntwork-Rodriguez S, Jiang Z, Solanoy H, Sengupta Ghosh A, Wang B, Kaminker JS, Huang K, Eastham-Anderson J, Siu M, : Dual leucine zipper kinase-dependent PERK activation contributes to neuronal degeneration following insult. Elife 2017, 6. 10.7554/eLife.20725 PMC5404924 28440222

[81] Wang C, Xiong M, Gratuze M, Bao X, Shi Y, Andhey PS, Manis M, Schroeder C, Yin Z, Madore C, : Selective removal of astrocytic APOE4 strongly protects against tau-mediated neurodegeneration and decreases synaptic phagocytosis by microglia. Neuron 2021, 109:1657–1674 e1657. 33831349 10.1016/j.neuron.2021.03.024 PMC8141024

[82] Shi Y, Yamada K, Liddelow SA, Smith ST, Zhao L, Luo W, Tsai RM, Spina S, Grinberg LT, Rojas JC, : ApoE4 markedly exacerbates tau-mediated neurodegeneration in a mouse model of tauopathy. Nature 2017, 549:523–527. 28959956 10.1038/nature24016 PMC5641217

